# *IDH* mutations are rare events in SHH medulloblastoma

**DOI:** 10.1007/s00401-025-02961-9

**Published:** 2025-11-24

**Authors:** Alicia Fürst, Viktoria Ruf, Christian Fiedler, Stefan Rutkowski, Martin Sill, Andrey Korshunov, Nicolas U. Gerber, Stephan Frank, Jürgen Hench, Ulrich Schüller

**Affiliations:** 1 Department of Pediatric Hematology and Oncology, University Medical Center Hamburg-Eppendorf, Hamburg, Germany; 2 Research Institute Children’s Cancer Center Hamburg, Hamburg, Germany; 3 Institute of Neuropathology, University Medical Center Hamburg-Eppendorf, Hamburg, Germany; 4 Center for Neuropathology and Prion Research, Faculty of Medicine, Ludwig-Maximilians-University Munich, Munich, Germany; 5 Hopp Children’s Cancer Center Heidelberg (KiTZ), Heidelberg, Germany; 6 Division of Pediatric Neuro-Oncology, German Cancer Consortium (DKTK) and German Cancer Research Center (DKFZ), Heidelberg, Germany; 7 National Center for Tumor Diseases (NCT) Heidelberg, A Partnership Between DKFZ and Heidelberg University Hospital, Heidelberg, Germany; 8 Department of Oncology, University Children’s Hospital, Zurich, Switzerland; 9 Institute for Pathology and Medical Genetics, University Hospital Basel, Basel, Switzerland

Hotspot mutations in *Isocitrate dehydrogenase 1* (*IDH1)* or *IDH2* have been discovered as a frequent oncogenic event in gliomas in 2009 [8]. According to the most recent version of the WHO classification of brain tumors from 2021, such mutations define what is now called astrocytoma, IDH-mutant and oligodendroglioma, IDH-mutant and 1p/19q-codeleted [3]. Approximately 85% of these mutations are IDH1:p.R132H mutations with other amino acid exchanges at codon 132 of IDH1 and codon 172 of IDH2 being rare. Of note, no other brain tumor entity has been reported so far to carry any recurrent IDH mutations. Here, we describe a series of 16 Sonic hedgehog (SHH) medulloblastomas (Fig. 1a) with six novel and ten previously published cases [2, 4, 6]. Thirteen of these cases carry an IDH1:p.R132C mutation, with the remaining three cases harboring an IDH1:p.R132L (case 4), IDH1:p.R132S (case 6), or IDH2:p.R172M (case 1) mutation (Fig. 1a). Patients have a median age of 26.5 years (range: 6–50). Apart from expected mutations in genes encoding for components of the SHH pathway, such as *PTCH1, SMO*, or *SUFU*, two cases (12.5%) displayed a homozygous, and one case (6.3%) displayed a heterozygous deletion of *CDKN2A/B*, a feature that is well known from high-grade IDH-mutant astrocytomas but not from medulloblastomas. In fact, only 9/2432 analyzed medulloblastomas of all types (0.2%) revealed a homozygous deletion of *CDKN2A/B*. Dimensionality reduction of global DNA methylation data confirms that all 16 cases display the highest similarity to SHH medulloblastoma (Fig. 1b). Furthermore, at least nine cases demonstrate a relatively similar DNA methylation profile (Fig. 1c), which is in line with the separate methylation class called MB_SHH_IDH that is used as a reference in a recently launched random-forest-based brain tumor classifier [5]. Copy number data that were inferred from the global DNA methylation data revealed gains in chromosome 3q as a frequent event, similar to what is observed in other SHH medulloblastomas of children or adults (SHH_3 and SHH_4, Suppl. Figure 1–2). Global DNA hypermethylation as it occurs in IDH-mutant astrocytomas [7] is significantly increased in IDH-mutant medulloblastoma compared to the other four SHH subtypes, whereas overall survival was comparable to the entire group of SHH medulloblastomas (Suppl. Figure 3). Histological analyses of such cases revealed typical features of medulloblastoma with densely-packed embryonal tumor cells expressing synaptophysin and p75 (Fig. 1e–h). OTX2 is not expressed, OLIG2 is expressed in a relatively low number of cells, and proliferative activity is high (Fig.1i–k).

**Fig. 1:**
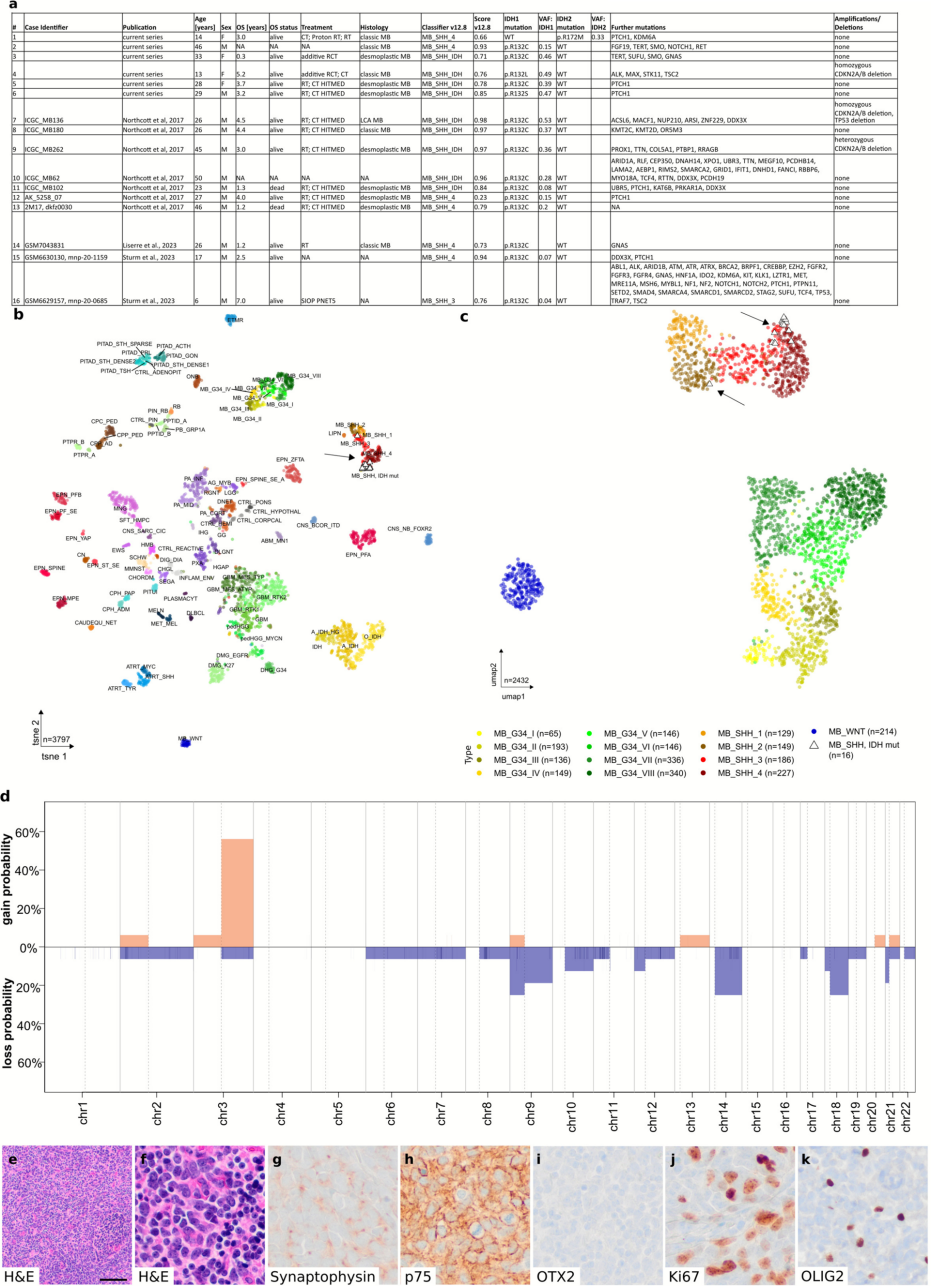
Overview of 16 medulloblastoma cases carrying IDH mutations. Details are summarized in **a**. Global DNA methylation analyses based on 3793 brain tumor reference cases [1] show these cases (marked by arrows) to fall into the class of SHH medulloblastoma (**b**). A more detailed analysis includes a selection of 2432 publicly available cases of WNT, SHH, Group 3, and Group 4 medulloblastomas (**c**). A cumulative copy number plot reveals gains on chromosome 3q (**d**), and histology shows typical features of SHH medulloblastoma (**e**–**k**). Scale bar in **e** corresponds to 100 µm in **e** and to 20 µm in **f**–**k**

Our report demonstrates that recurrent IDH mutations in the central nervous system may occur beyond astrocytomas and oligodendrogliomas. At a frequency of approximately 5%, IDH mutations are rare events in SHH medulloblastoma. While it is tempting to speculate that IDH inhibitors may represent a therapeutic option for patients with IDH-mutant SHH medulloblastomas, future studies exploring the functional relevance as well as the predictive and prognostic value of this molecular alteration in larger cohorts are warranted.

## Supplementary Information

Below is the link to the electronic supplementary material.

Supplementary file1 (PDF 20314 KB)

Supplementary file2 (PDF 183 KB)

Supplementary file3 (PDF 533 KB)

## Data Availability

DNA methylation data are available via GEO accession number GSE307314.
