# Supplementary material for: IDH mutations are rare events in SHH medulloblastoma
Source: Acta Neuropathol. 2025 Nov 24;150(1):55. doi: 10.1007/s00401-025-02961-9 (PMC12644213; doi:10.1007/s00401-025-02961-9)
Supplement: Supplementary file 1 — Supplementary file1 (PDF 20314 KB) [file 401_2025_2961_MOESM1_ESM.pdf]

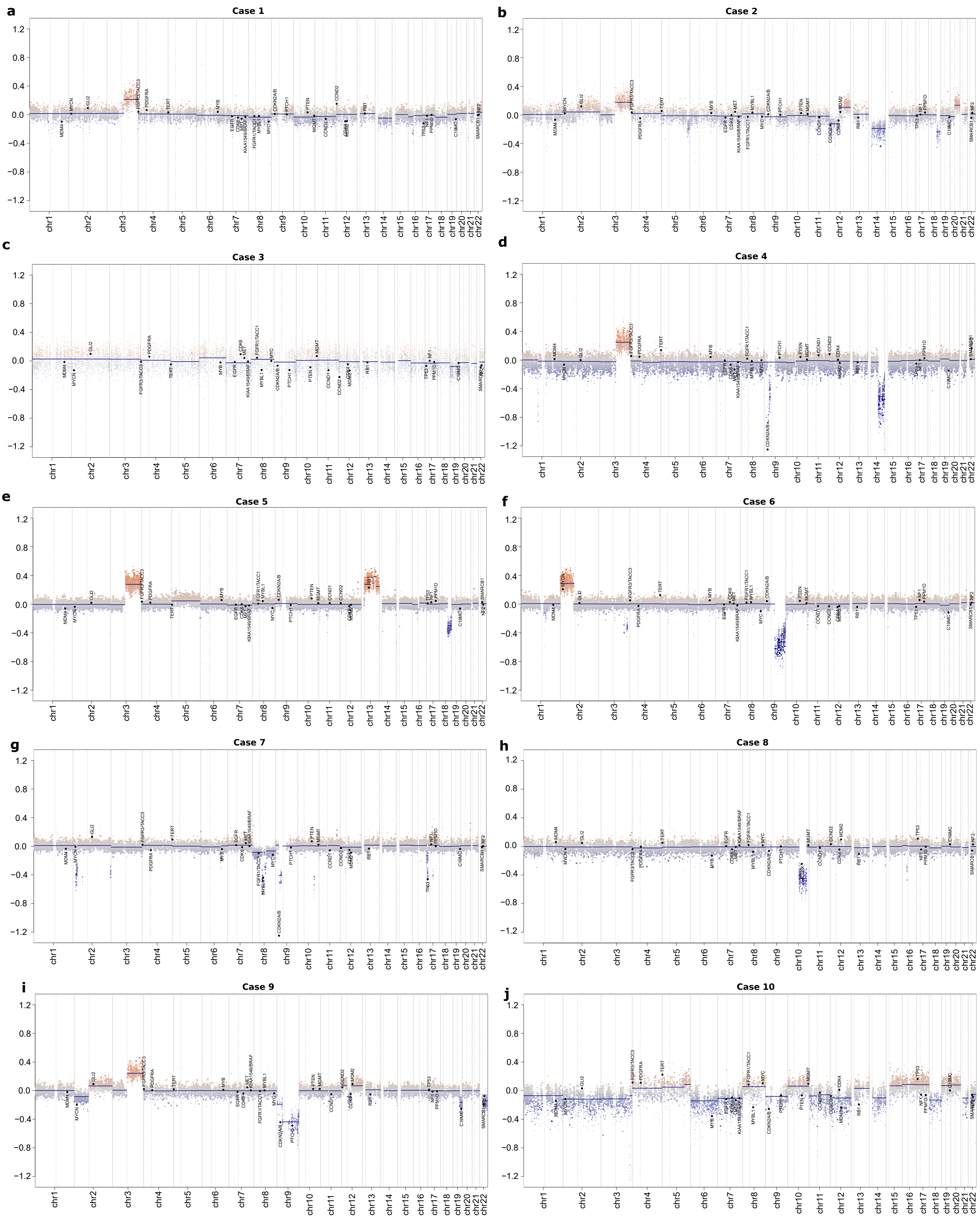

a
Case 1
b
Case 2
c
Case 3
d
Case 4
e
Case 5
f
Case 6
g
Case 7
h
Case 8
i
Case 9
j
Case 10

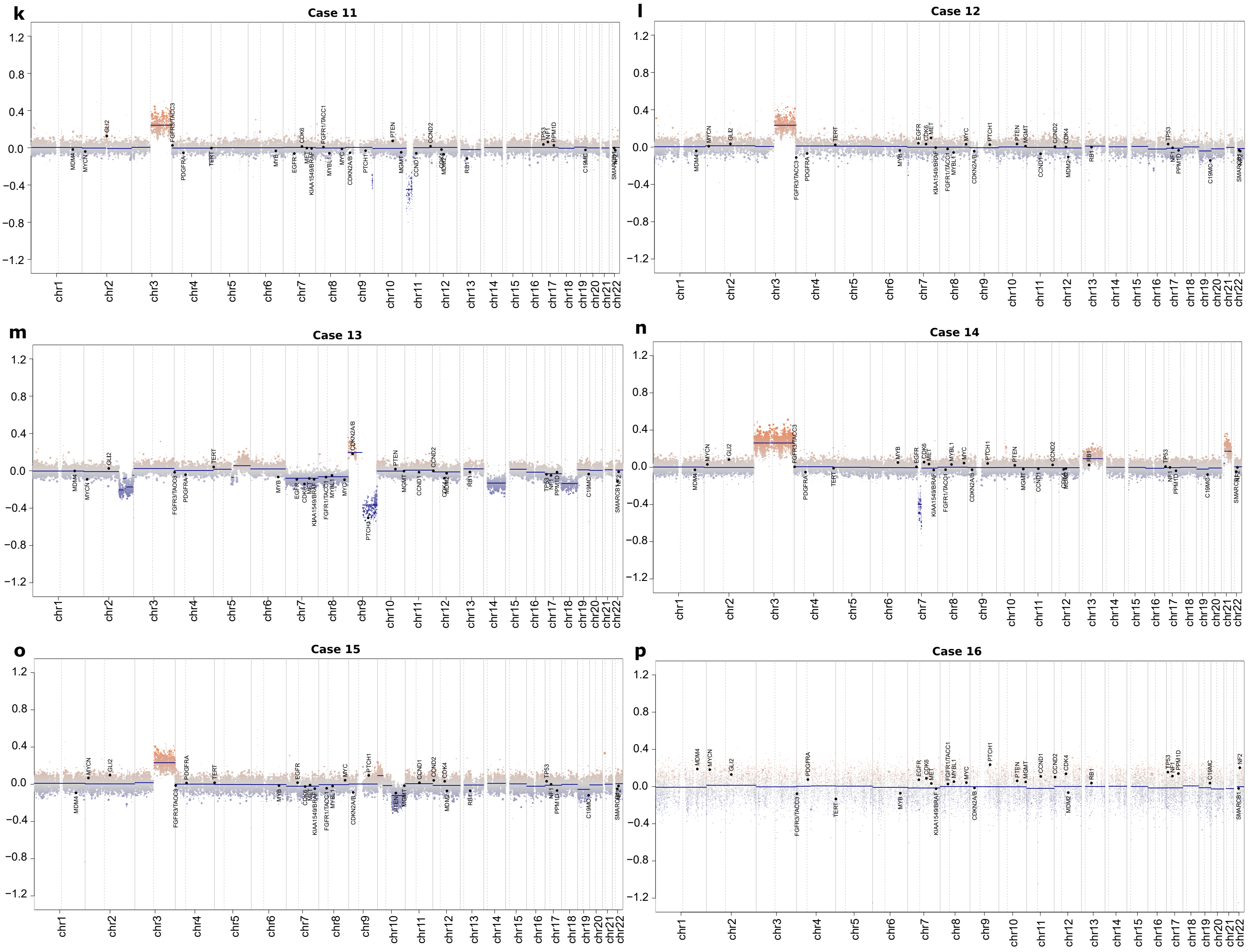

Supplementary Figure 1: Copy number variation plots of *IDH* mutated medulloblastoma. Nine samples display a gain of chromosome 3q, while in three cases a focal deletion of *CDKN2A/B* is observed.
