# Supplementary material for: IDH mutations are rare events in SHH medulloblastoma
Source: Acta Neuropathol. 2025 Nov 24;150(1):55. doi: 10.1007/s00401-025-02961-9 (PMC12644213; doi:10.1007/s00401-025-02961-9)
Supplement: Supplementary file 2 — Supplementary file2 (PDF 183 KB) [file 401_2025_2961_MOESM2_ESM.pdf]

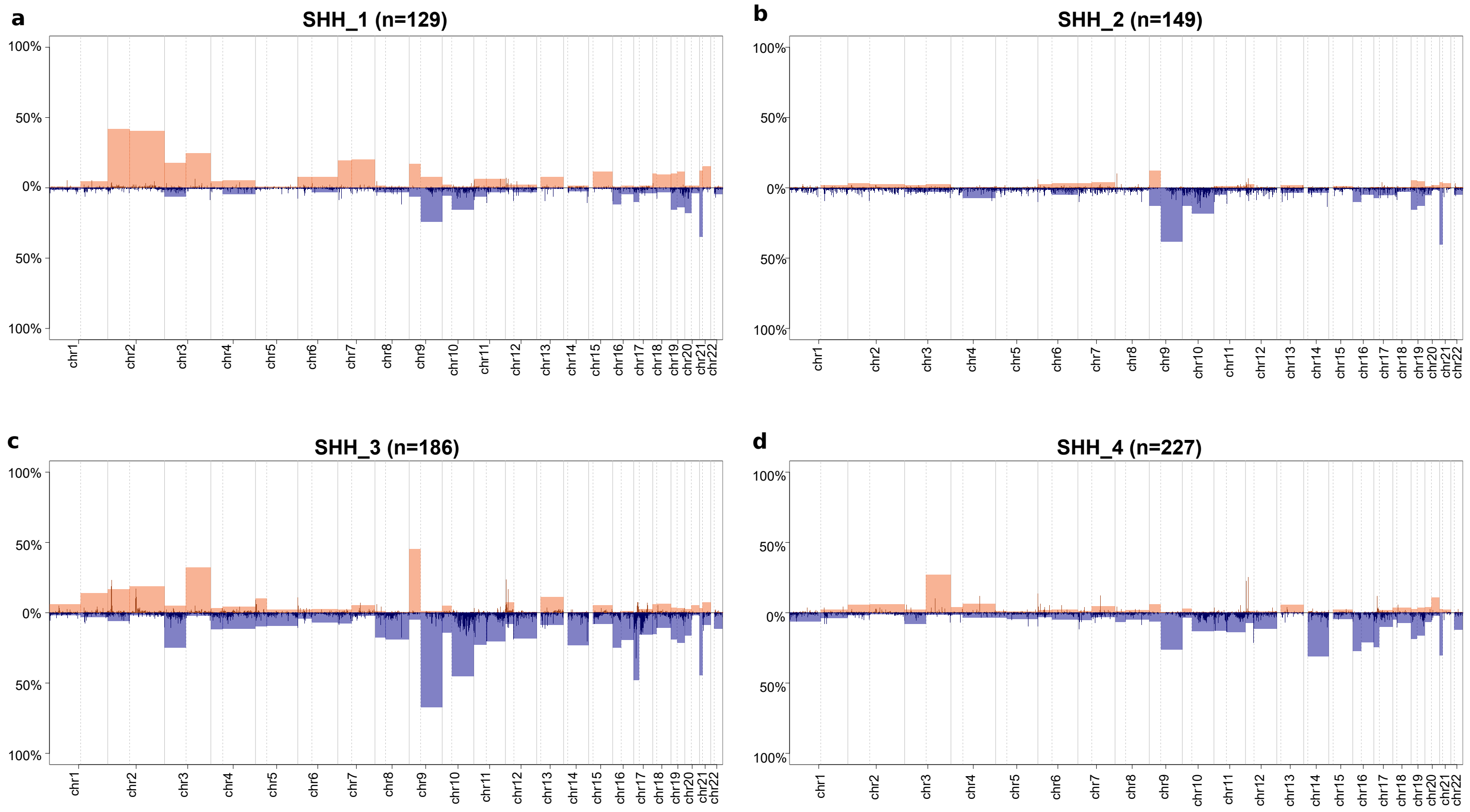

Supplementary Figure 2: Cumulative copy number variation profiles of four SHH subtypes. For each subtype, recurrent chromosomal gains (red) and losses (blue) are displayed as cumulative frequencies across all samples. Focal amplifications (thin dark-red lines) and focal deletions (thin dark-blue lines) are indicated separately.
