# Supplementary material for: IDH mutations are rare events in SHH medulloblastoma
Source: Acta Neuropathol. 2025 Nov 24;150(1):55. doi: 10.1007/s00401-025-02961-9 (PMC12644213; doi:10.1007/s00401-025-02961-9)
Supplement: Supplementary file 3 — Supplementary file3 (PDF 533 KB) [file 401_2025_2961_MOESM3_ESM.pdf]

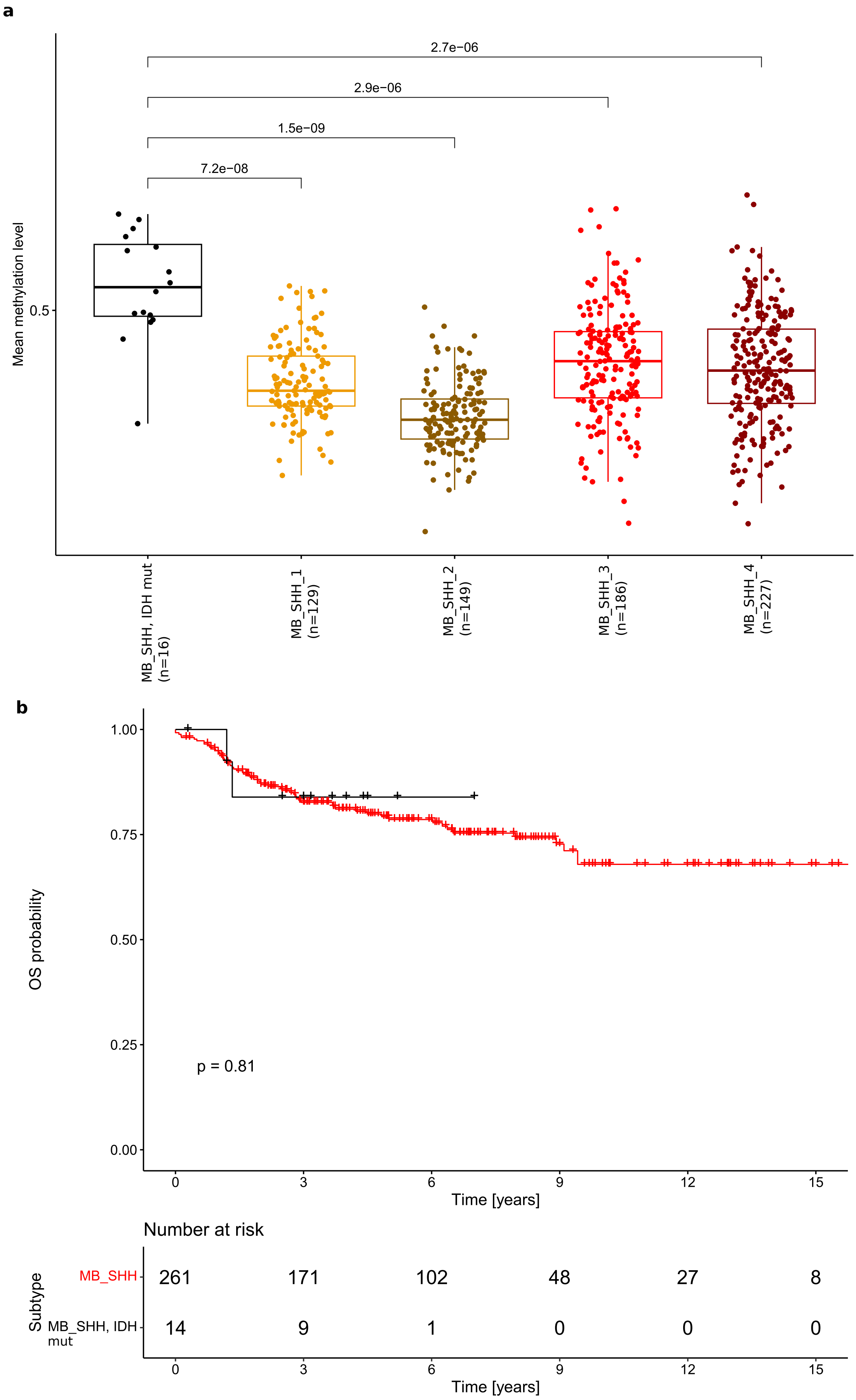

Supplementary Figure 3: Mean global DNA methylation and overall survival of IDH mutant medulloblastoma. For the IDH mutant medulloblastoma and 4 known methylation subtypes of medulloblastoma the mean global DNA methylation of 450k CpG sites were determined (a). The overall survival of the IDH mutant medulloblastoma was compared to the other SHH medulloblastoma (b).
